# Analysis of lead, arsenic and calcium content in the hair of children with autism spectrum disorder

**DOI:** 10.1186/s12889-020-08496-w

**Published:** 2020-03-23

**Authors:** Joanna Fiłon, Jolanta Ustymowicz-Farbiszewska, Elżbieta Krajewska-Kułak

**Affiliations:** 1 Department of Integrated Medical Care, Medical University of Bialystok, Marii Sklodowskiej-Curie 7A, 15-096 Bialystok, Poland; 2 Department of Hygiene, Epidemiology and Ergonomics, Medical University of Bialystok, 15-089 Bialystok, Poland

**Keywords:** Heavy metals, Lead, Arsenic, Calcium, Children, ASD, Hair

## Abstract

**Background:**

Explanation of the pathogenesis and treatment of autism spectrum disorders (ASD) is one of the most significant challenges for scientists today. It is believed that a major pathogenetic factor of this condition is epigenetic changes caused by environmental factors, including toxic metals (cadmium (Cd), lead (Pb), mercury (Hg), aluminium (Al), and arsenic (As)). The nervous system may also be affected by deficiencies of both micro- and macroelements (e.g. calcium (Ca), zinc (Zn)). The aim of the study was to analyze the concentrations of Pb, As, and Ca in the hair of children with ASD and a control group.

**Methods:**

The materials for the study comprised hair samples collected from 30 children diagnosed with ASD (case group) and 30 children randomly selected from the general population of Bialystok and surrounding region (control group). Concentrations of Pb, As, and Ca were tested with electron microscopy scanning method. Next, the content of the analyzed elements in the hair was assessed as well as their impact on autism development in the children and the mutual interactions between them. The obtained results were statistically analyzed with Statistica PL 12.5., using the Mann-Whitney U test, and Spearman correlation coefficient.

**Results:**

Mean Ca level in the hair of the case group was lower than the mean level of this element in the control group. Mean As and Pb concentration in the hair of children with ASD was statistically significantly higher than the mean concentration of this element in the hair of children without neurological disorders. Statistically insignificant weak positive correlations between Ca and As content and negative between Ca and Pb in the hair of children from the case group were noted. Also, statistically significant mean positive correlations between Pb and As were observed.

**Conclusions:**

In this small study, according to the observations, children diagnosed with ASD suffer from Ca deficiency and toxic metal overload (As and Pb). These abnormalities may play the main role, as an environmental factor, in the pathogenesis of the analyzed disorder.

## Background

Autism Spectrum Disorder (ASD) is one of the most common neurodevelopmental impairments (in 2014 it was diagnosed in 1 out of 68 children) and is four times more common in boys than girls [1] (https://www.cdc.gov/ncbddd/autism/data.html). This ratio is higher in those with a mild course of the disorder compared to individuals with an acute course of autism. Over 50% of children with autism have an intellectual disability, while every third child develops epilepsy and every second child suffers from speech disorders. Apart from intellectual deficits, ASD may also be accompanied by metabolic disorders, motor organ development abnormalities, and chronic somatic diseases [1] (https://www.cdc.gov/ncbddd/autism/data.html).

In previous years, an increase in the number of diagnosed ASD cases has been observed, which allows to hypothesize that chemical contamination in the environment influences these sorts of developmental disorders [2–4]. Studies indicate the neurotoxic effect of numerous substances, including heavy metals (on mitosis, cell differentiation, synapse formation, oxidative stress, endoplasmic reticulum stress, and essential metalloprotein disruption, apoptotic processes, level of neurotransmitters). Lead (Pb) and arsenic (As) are well-established neurotoxicants known to cross the blood–brain barrier and affect neurodevelopment [1, 5–7]. To give an example, Pb impairs intellectual development, causes behavioral disorders and motor hyperactivity. It can also have adverse effects on the health of children, causing behavioral and neurological problems, and a reduction in IQ scores [1, 5, 6, 8–11]. Similar effects can be observed in the case of As. Arsenic exposure significantly affects brain morphology, resulting in gliosis, neuronal degeneration, a decrease in cognitive abilities, attention, comprehension, language skills, and reduces intelligence quotient (IQ) scores [1, 5, 6, 11–14]. The high levels of heavy metal antagonists (e.g. Ca) in the body act protectively to a certain degree. However, a Ca deficiency can further increase the toxic effect of Pb and As [7, 8, 15].

ASD is a neurodevelopmental disorder, this suggests a possible role of heavy metals in its underlying cause (induction). However, investigations on associations between ASD and neurotoxic heavy metals are inconclusive. Over the last decades, numerous studies have reported a relationship between ASD and Pb and As exposure [5, 11–13, 16–24]. However, there are also some reports that conclude that heavy metals exposure is not a risk factor for ASD [6, 14, 25–29]. Therefore, an accurate evaluation of the relationship between ASD and heavy metals needs more specific research. This study is the first in Poland and may be an important contribution to the body of knowledge on the subject of metal concentration in the hair of children with ASD.

The aim of the study was to analyze Pb, As, and Ca concentrations in the hair of children with ASD and a control group.

## Methods

### Materials

The materials for the study were hair samples collected from children from an urban agglomeration in Poland. The study group comprised 30 children diagnosed with ASD (25 boys and 5 girls) aged from 2 to 8 years (mean age 5.250 ± 1.586 years), attending randomly selected therapeutic kindergartens in Bialystok. The control group consisted of 30 neurotypical children (25 boys and 5 girls; children who have not been diagnosed with neurological disorders) aged from 2 to 8 years (mean age 5.094 ± 1.510 years) randomly selected from the general population of Bialystok and the surrounding region.

### The procedure of preparing samples and testing element content

The hair of both groups, ASD cases and control, was taken from 6 different areas of the occipital part of the head. Each hair sample, weighing 0.2–0.5 g, consisted of 3 cm long sections (cut at the scalp). If hair was longer, only the last 3 cm from the scalp was examined.

Prior to sample analysis, the hair samples were degreased with chloroform, p.a. grade, three times and next rinsed in redistilled water and dried at 50–60 °C. Further rinsing was performed in a mixture of anhydrous ethanol, p.a. grade, and anhydrous acetone, p.a. grade, mixed in a ratio of 1:1, and then dried at laboratory temperature.

Concentrations of Pb, As, and Ca were tested with electron scanning microscopy on a Hitachi TM − 3000 apparatus with X-ray microanalyzer EDS.

An assessment of the analyzed element content in the hair, their impact on autism development in children, and dependencies between the concentrations of the analyzed trace elements in the hair was performed.

### Quality control

The accuracy of the method was verified on certified reference material NCS ZC81002 Human Hair (China National Analysis Center). The procedure was analogical to the one applied in testing the analyzed samples. Six tests of the concentration levels of the studied elements in the reference material were performed and the results were compared with the values provided in the certificate.

Standard solutions containing the analyzed elements in proper concentrations had been prepared for each batch. Based on the standard solutions, a calibration chart was compiled for reading the test results.

### Ethics, consent and permissions

The parents or legal guardians of each child signed a written protocol consent to participate in the study.

The study was approved by the Bioethics Committee of the Medical University of Bialystok (resolution no. R-I-002/18/2015).

### Statistical analysis

The results obtained for each child were averaged and statistically analyzed using StatSoft, Inc. (2014) software STATISTICA (data analysis software system), version 12.5. Statistical analysis of the results included calculating mean values, standard deviation, medians, and the minimum and the maximum of the analyzed elements in the hair. Prior to statistical analysis, an assessment of normal distribution with the Shapiro-Wilk test and the Kolmogorov-Smirnov test was performed. Next, variances of homogeneity were evaluated using the F-test and Levene’s test. The distribution of variables differed from normal distribution, therefore the non-parametric Mann-Whitney U test was used. Spearman correlation coefficient (R) was used to evaluate the strength of correlations between the analyzed variables. Statistical significance was considered at *p* ≤ 0.05.

## Results

Table 1 presents a comparison of mean levels of concentration, medians, scopes and standard deviations of selected trace elements in the hair of autism spectrum children and those without neurological disorders.

**Table 1:** Levels of particular trace elements in children’s hair (mg·kg^− 1^)

		Case group/*n* = 30	Control group *n* = 30	*p*
Ca	M ± SD	254.7 ± 91.96	312.8 ± 86.89	*p* = 0.002
	Median	219.6	290.8	
	Range	149.5–458.1	146.1–543.7	
As	M ± SD	0.216 ± 0.09	0.061 ± 0.03	*p* < 0.001
	Median	0.196	0.058	
	Range	0.051–0.499	0.004–0.133	
Pb	M ± SD	6.028 ± 0.69	3.415 ± 1.207	*p* < 0.001
	Median	5.793	3.822	
	Range	5.178–7.618	0.500–4.490	

Mann-Whitney U test was used; *p* < 0.05 (statistically significant)

*M* mean, *SD* standard deviation

**Fig. 1:**
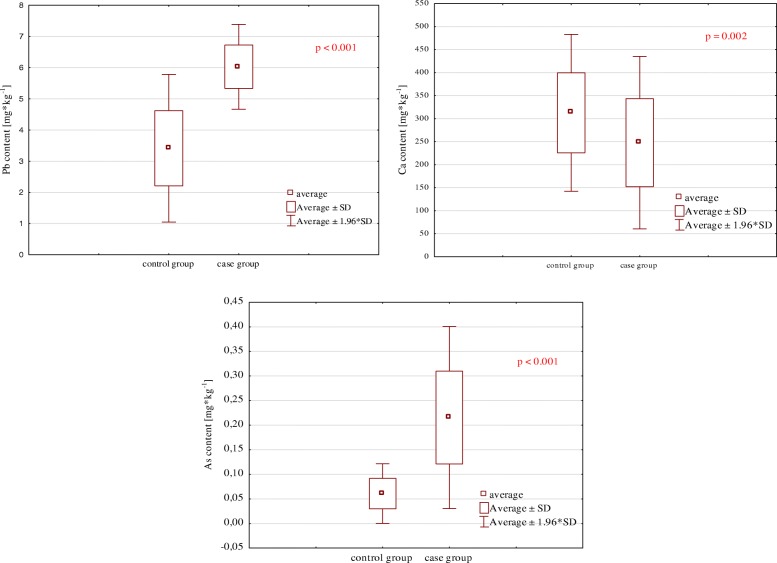
Relationships between the levels of the studied elements in the hair of ASD cases and the control group (mg kg^− 1^)

Mean Ca level in the hair of children with ASD (254.7 ± 91.96 mg kg^1^) was lower than the mean level of this element in the control group (312.8 ± 86.89 mg kg^− 1^) (statistically significant differences, *p* = 0.002). Mean As concentration in the hair of children with ASD was 0.216 ± 0.09 mg kg^− 1^ and was statistically significantly higher (*p* < 0.001) than the mean concentration of this element in the hair of children without neurological disorders (0.061 ± 0.03 mg kg^− 1^). According to observations, mean Pb concentration in the hair of children with ASD was 2-fold higher compared to mean Pb concentration in the control group (6.028 ± 0.69 mg kg^− 1^ and 3.415 ± 1.207 mg kg^− 1^, respectively) (statistically significant differences, *p* < 0.001) (Table 1, Fig. 1).

We found weak negative correlations between the levels of the analyzed metals. In the hair of children with ASD Ca was negatively correlated with Pb (*r* = − 0.18, *p* > 0.05) and positively with As (*r* = 0.11, *p* > 0.05). Statistically significant moderate positive correlations between Pb and As (*r* = 0.36, *p* < 0.05) in the hair of children with ASD were observed (Table 2).

**Table 2:** Correlation between the levels of the studied elements in the hair of the case group and the control group

		Case group			Control group		
		As	Ca	Pb	As	Ca	Pb
Case group	As	–	0.11	0.36*			
	Ca	0.11	–	−0.18			
	Pb	0.36*	−0.18	–			
Control group	As				–	−0.22	−0.08
	Ca				−0.22	–	− 0.15
	Pb				−0.08	−0.15	–

Spearman correlation coefficient was used * statistically significant (*p* < 0.05)

## Discussion

There is a list of 201 industrial chemical agents that have a neurotoxic effect on humans (including Pb and As). They may cause autism and other disorders such as attention deficit, mental retardation, and cerebral palsy [1, 16, 17, 23, 24].

Arsenic (As) is an element that is common in nature and is toxic. It is absorbed into the body primarily through the digestive tract and the respiratory system. In the human body, As compounds may inhibit the activity of over 200 enzymes. The effects of chronic As intoxication include neurotoxic effects in the central and the peripheral nervous system. The symptoms may manifest as sensory changes, muscle sensitivity, sensation of stinging and tingling (paresthesis), weakness, progressing muscle flaccidity, and polyneuropathy with sensory loss. A particularly unfavorable impact is exerted on the central nervous system, and in the case of pregnant women on the development of the fetal nervous system, since it crosses the blood-placenta barrier and accumulates in fetal epithelial tissue in the early stages of pregnancy. In children whose mothers were exposed to As compounds, the following symptoms are observed: difficulty with learning, memory, concentration, cognitive and behavioral disorders. Children exposed to this element may also present the symptoms of retarded development or inhibited physical and mental development [1, 5, 6, 13, 23, 29–35].

Lead (Pb) is one of the most dangerous metals due to its common occurrence in the environment. It is highly toxic, has the ability to easily cross biological barriers, and accumulates in the internal organs [16, 17, 36]. Pb may affect the functions of both the central and peripheral nervous system as well as the senses. Numerous studies have confirmed that excessive exposure to Pb results in, among others, convulsions, changes in brain functions, encephalogram changes as well as acute encephalopathy and other brain disorders. In cases when Pb concentration in the plasma is over 30 mg·L^− 1^, gradual changes in behavior and cognitive functions may occur manifesting as irritability, sensory disorders, apathy, impaired visual-motor coordination, and longer time of responding to stimuli [1, 5, 6, 8, 15, 22–24, 29, 37–40].

The nervous systems of fetuses and small children show higher sensitivity to Pb intoxication compared to adults. This results from a greater susceptibility of their brains to disorders (especially in the period of fast growth). Functional changes of the brain develop in children at over 10 mg Pb/10^− 1^ L of blood causing most of all decreased IQ. Also, Pb in children leads to disorders of cognitive functions, difficulty learning (mostly reading, learning languages, and mathematics), lack of concentration, impairment of a short-time memory, recurring epileptic seizures, irritability, hyperactivity as well as impairment of speech, vision and even personality (e.g. aggression) [6, 15, 31, 32, 37–42].

In our research, mean As and Pb content in the hair of children with ASD was statistically significantly higher compared to the mean content of these elements in the hair of children from the control group (Table 1, Fig. 1). Numerous studies have confirmed that heavy metals play a crucial role in the development of autism spectrum disorders [1, 5, 11–13, 16–22, 24]. Yasuda et al. [16, 17] demonstrated increased Pb concentrations in 4.8% of children with ASD and As in 2.6% (maximal Pb concentration amounted to 24.9 ppm, while As 1.7 ppm). Fido and Al-Saad [25] also observed a significantly higher Pb concentration in 4–7 year old children with autism compared to normal individuals (6.75 vs 3.20 mg·kg^− 1^). Similar Pb and As content were found in the hair of ASD children from different parts of the world, Saudi Arabia [11, 12, 18, 43], Kuwait [25], Oman [19], India [20], Egypt [21, 22], Italy [26], and the USA [5, 29, 44].

A study conducted on a large group of Mexican children showed a positive correlation between Pb concentration in the blood and increased severity of overactivity symptoms. Pb content showed no correlation with attention disorder, which is characteristic of full blown ADHD (attention-deficit hyperactivity disorder) [45]. Meta-analysis of 33 studies published in the years 1972–2010 conducted on 10,232 children showed a positive dependency between Pb concentration in the blood and hair and overactivity and attention disorders [41]. According to observations, low Pb concentration in the blood (< 3.4 mg·L^− 1^) may be a factor that additionally increases the risk of genetically conditioned ADHD. It was demonstrated that exposure to Pb causes cognitive deficits similar to those observed in ADHD children as well as impaired attention and cognitive function. Also, studies conducted on children indicated that Pb impairs the processes of inhibiting response and attention in school-age children. It has also been proven that in the case of blood Pb concentration > 2.0 mg·L^− 1^ the risk of developing ADHD increases over four times [46].

Numerous clinical control studies have proven increased levels of one or more toxic metals in urine, blood, hair, nails, teeth, and brain samples in autism spectrum children compared to children showing normal development [24, 47]. Al-Ayadhi [18] and Blaurock- Busch et al. [11, 12] observed significantly higher concentrations of heavy metals (Pb and As) in the hair of children with ASD compared to healthy children. Adams et al. [27, 47] also demonstrated that children with autism had higher Pb and As concentrations in urine (by 72 and 21%, respectively) and in the hair (by 30 and 20%, respectively) compared to healthy children.

There is increasing evidence that children with ASD have a considerable disability to process and eliminate toxins from the body [1, 5, 6, 14, 43]. Toxin accumulation leads to increased activity of free radicals in the body, which in turn affects the structure of the nervous system. It is worth noticing that detoxification and eliminating heavy metals involves their conjugation with glutathione, whose concentrations are considerably lower in ASD individuals [19].

Modern people’s exposure to the risk is complex. Mutual interactions may occur between particular elements. Toxic metals affect trace element absorption, while the interaction between essential elements and toxic metals affects threshold values and toxicity effects. Toxic metals may interact metabolically with nutritionally essential metals [11, 12, 18]. Resorption of some ions (Ca^2+^, Mg^2+^, Fe^2+^, Zn^2+^ or Cu^2+^) from the digestive tract is inhibited by Pb, which is indicative of absorption competitiveness of these elements in the intestines. Also, Pb impairs the metabolism of minerals and causes multisystemic toxic effects through the inactivation of many enzymes (it binds to sulfhydryl, carboxyl and amino groups of aminoacids and proteins), leads to impaired effects of vital cations (Ca, Zn and iron (Fe)), abnormal oxidation-reductive condition of cells, impaired structure of cellular membrane and receptor functions. Increased Pb content enhances Ca elimination, while decreased Ca content in the diet causes a higher accumulation of this metal, especially in bones [8, 15, 38, 39]. A Ca deficiency may additionally strengthen the toxic effects of Pb and affect cognitive and behavioral development in children. A significant converse dependency between Ca intake with diet and Pb concentration in the blood was observed as early as in the 1990s in 3000 American children in the NHANES II study [7].

According to our research, we found weak negative correlations between the levels of the analyzed metals. In the hair of children with ASD, Ca was negatively correlated with Pb and positively with As. Statistically significant moderate positive correlations between Pb and As in the hair of children with ASD were observed (Table 2). The antagonistic effects of toxic metals and bioelements have been confirmed by a number of studies [10–12, 16, 18, 20, 27, 47–50]. The mechanism through which Ca impairs As absorption and conversely has not been fully understood so far. Ca is able to participate in the inhibition and absorption of As through competitiveness connected with a common binding site with proteins in the intestines [16, 17, 33, 47]. Heavy metals, e.g. Pb and As, have a very similar toxic effect, which in turns leads to the increased severity of intoxication symptoms with concurrent exposure to more than one metal [1, 5, 6, 24, 33].

Ca is a mineral necessary for the normal functioning of the whole body. It regulates the nervous system by improving the transmission of nerve impulses (it is a transmitter of arousal states in nerve synapses). Low concentrations of Ca in the body cause the following symptoms: irritability and excitability, nervousness, oversensitivity and anxiety states. Chronic Ca deficiency in children may lead to mental disorders [7, 51].

In our study, mean Ca content in the hair of children in the ASD case group was lower than the mean content of this element in the control group (statistically significant differences) (Table 1, Fig. 1). Many authors have also observed significantly lower Ca content in children with autism compared to healthy children. This suggests that children with ASD are susceptible to Ca deficiency [16–18]. The presented results have been reflected in studies by Japanese researchers who noticed a Ca deficiency in approximately 6% of children. A considerable deficiency of this element has been diagnosed mainly in children under 10 years old. The minimal Ca concentration in this group amounted to approximately 70 ppm [16, 17]. Apart from Ca deficiency, many children with autism also had a deficiency of other elements, including Zn, Mg, Mn or Cu [12, 16, 17, 19, 49]. It was also demonstrated that children with autism had significantly lower Ca content in the hair (by 20%) compared to normal children [27].

Despite a number of studies and analyses, autism is still not understood by scientists, because no specific factor causing this condition has been found. Each experiment provides new valuable data but fails to deliver an explanation for symptom occurrence.

### Limitations

In this study there are several limitations that could be solved in future studies. The main limitation of our work is the small number of cases and the strong regional focus of this study. Samples were taken only from one mid-size city in Poland in a region with rather low pollution levels. The narrow group of only 30 cases of diagnosed children is also too population-specific and probably is not representative of neurodevelopmental disorders in the whole country. The limited number of cases also does not provide adequate statistical confidence. But our work is the first (of its kind) in Poland and hopefully could be the basis for future studies that will have greater (country) range and contribute to the global picture of ASD.

Secondly, there is a very limited number of studies in Europe on the subject of metal concentration in the hair of children with ASD. In the case of research into relationships between metals content in the hair and neurodevelopment disorders, tests should be carried out within larger social groups (preferably international) to avoid falsifying the results through any local influence.

Thirdly, the study did not plan to collect other data on the study subjects. The influence of sociodemographic and environmental factors (medical history, metabolic abnormalities, diet, environmental contamination) on the content of these metals in the hair was also not taken into account. The research results lack information about the exposure of mothers before pregnancy and during pregnancy as well as infants / children at a very early age.

Fourthly, cosmetic procedures have a significant impact on the content of elements determined in the hair. In our research, we did not include the types of cosmetics (e. g. shampoos) used by the children. We only assumed that the examined group consisted of rather small children so they probably use only basic hygiene products, which is not necessarily true. Thus, future studies should consider the relationship between children’s cosmetics and contamination in hair.

Fifthly, there are no generally applicable standards for the content of trace elements in the hair of any people (healthy or with any diseases).

Our results are informative, add to the literature, and might make a contribution to the body of knowledge on the subject of trace elements in ASD. More research is needed about the relationships between ASD and heavy metals exposure. Explanation of the pathogenesis and treatment of neurological disorders is one of the greatest challenges for scientists today.

## Conclusions

1. In this small study statistically significantly higher As and Pb content in the hair of children with ASD compared with the control group was observed.
2. Decreased Ca content in the hair of the case group children compared with the control group was demonstrated.
3. Notwithstanding evident limitations, the study suggests that abnormal concentrations of the analyzed elements may indicate a pathophysiological role of heavy metals and trace elements in the genesis of symptoms of autism spectrum disorders.

## Data Availability

The data and material are available upon reasonable request from the corresponding author. E-mail: joanna@umb.edu.pl

## Abbreviations

ADHD: Attention-Deficit Hyperactivity Disorder
Al: Aluminium
As: Arsenic
ASD: Autism Spectrum Disorder
Ca: Calcium
Cd: Cadmium
Fe: Iron
Hg: Mercury
M: Mean
Mg: Magnesium
Mn: Manganese
Pb: Lead
SD: Standard deviation
Zn: Zinc
